# Multidisciplinary management of bronchoesophageal fistula using adipose-derived stromal vascular fraction and platelet-rich plasma

**DOI:** 10.1016/j.vgie.2025.09.007

**Published:** 2025-10-09

**Authors:** Joris A. van Dongen, Roos E. Pouw, Metin Bülbül, Hans J.L. Kemming, J. Henk Coert, Richard van Hillegersberg

**Affiliations:** 1 Department of Plastic, Reconstructive, and Hand Surgery, University Medical Center Utrecht, Utrecht, the Netherlands; 2 Department of Gastroenterology, University Medical Center Utrecht, Utrecht, the Netherlands; 3 Department of Pulmonology, University Medical Center Utrecht, Utrecht, the Netherlands; 4 Department of Upper Gastrointestinal Surgery, University Medical Center Utrecht, Utrecht, the Netherlands

## Abstract

**Background and Aims:**

Bronchoesophageal fistula (BEF) is a rare but life-threatening adverse event following surgery. Fistulas result in respiratory adverse events, often requiring surgical repair, which is a high-risk procedure. Therefore, minimally invasive alternatives are needed. We aimed to use adipose-derived stromal vascular fraction (SVF) to treat BEFs.

**Methods:**

We present 2 BEF cases after minimally invasive esophagectomy and gastric conduit reconstruction. During the procedure, SVF was isolated and platelet-rich plasma (PRP) obtained. In 1 case with a tracheaesophageal fistula, simultaneous bronchoscopy and endoscopy were performed to localize and treat the fistula; in the second case, only gastroscopy was performed to treat a fistula from the esophagus to the right upper lobe. During the procedures, the epithelialized fistula tract was cleared using brushing and argon plasma coagulation, followed by injection of SVF-PRP around the fistula. Closure was obtained using a through-the-scope suturing system.

**Results:**

The patients recovered well, and an esophagram 4 weeks postprocedure showed no more signs of a fistula. Currently, 7 and 4 months postprocedure, respectively, both patients are on a normal oral diet and with restored pulmonary function.

**Conclusions:**

These cases demonstrate that SVF-PRP injection combined with endoscopic closure may offer a promising minimally invasive alternative for BEF treatment.

## Introduction

Bronchoesophageal fistula (BEF) is a rare (3%) but potentially life-threatening adverse event following thoracic surgical procedures.^1^ Current treatment options include surgical repair or endoscopic interventions such as stent placement, clipping, or injection of bioglue. Because long-term outcomes of these endoscopic techniques are often disappointing, surgical repair currently remains the most effective option.^2^^,^^3^ Unfortunately, not all patients are amenable for surgery because of their frail condition. Endoscopic techniques could be a solution.^4^ Recently, studies have shown promising results and improved closing rates with the use of adipose stromal vascular fraction (SVF) and platelet-rich plasma (PRP).^5^, ^6^, ^7^ Mechanically isolated adipose SVF consists of all nonadipocyte cell types such as adipose-derived stromal cells (ASCs), endothelial cells, immune cells, fibroblasts, as well as extracellular matrix. ASCs are believed to be one of the key cell types to produce a plethora of growth factors and cytokines that modulate immune response,^8^^,^^9^ act pro-angiogenic,^10^^,^^11^ and remodel the extracellular matrix to improve wound healing.^8^ The extracellular matrix in mechanically isolated SVF functions as a slow controlled-release scaffold of these bound paracrine factors.^8^^,^^9^ The addition of PRP stimulates ASCs present in SVF to increase the release of pro-regenerative factors.^12^

## Case Presentation

We present 2 cases with a BEF after esophagectomy: a 76-year-old man with a tracheaesophageal fistula and a 74-year-old man with a fistula to the right upper lobe. Both patients had infection problems, cough, inability for oral intake, and generalized weakness as a result their conditions.

## Stromal Vascular Fraction Isolation

**Figure 1:**
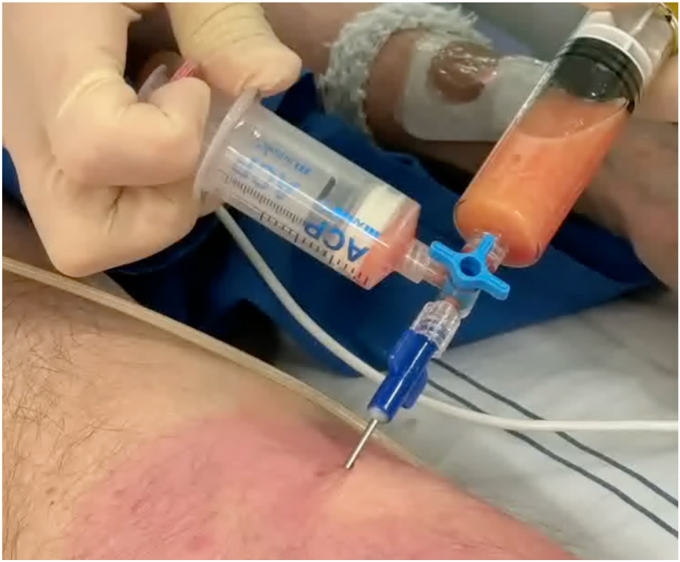
Liposuction of subcutaneous fat after infiltration with saline/adrenaline/lidocaine.

**Figure 2:**
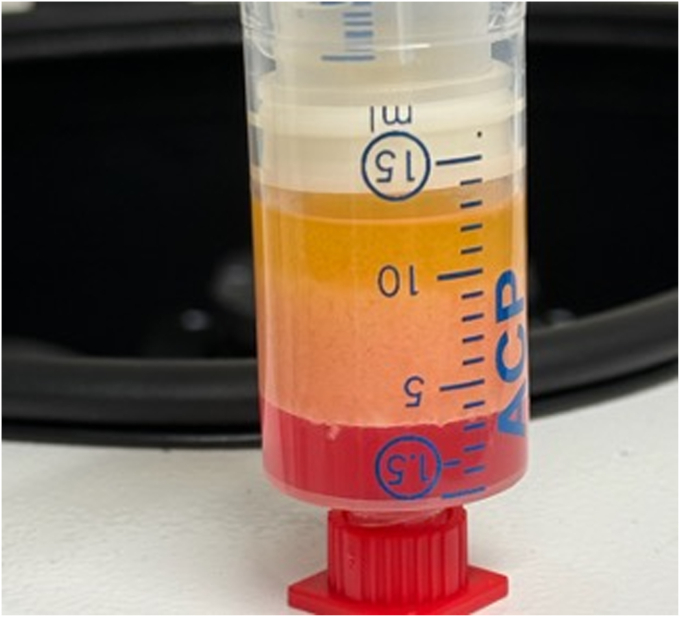
After centrifugation, 3 layers can be distinguished, from top down: oil (broken adipocytes), centrifuged fat, and infiltration fluid.

One procedure was performed with the patient under general anesthesia, and the other with the patient under propofol sedation. Subcutaneous adipose tissue was harvested (15 mL) and processed according to the fractionation of the adipose tissue procedure (Arthrex, Utrecht, the Netherlands) (Fig. 1).^13^^,^^14^ Briefly, infiltrated adipose tissue was harvested, centrifuged, fractionated (through a 1.4-mm Luer-to-Luer [Arthrex] lock connector), and centrifuged again. Three separate layers were formed: oil, SVF, and infiltration fluid. Oil and infiltration fluid were discarded (Fig. 2). Simultaneously, 15 mL of whole blood was obtained to create PRP. PRP (7 mL) was mixed with adipose SVF (1 mL) (Video 1, available online at www.videogie.org). Preparation of the injectate requires 30 to 45 minutes.

## Broncho- and Endoscopy Treatment of the BEF

**Figure 3:**
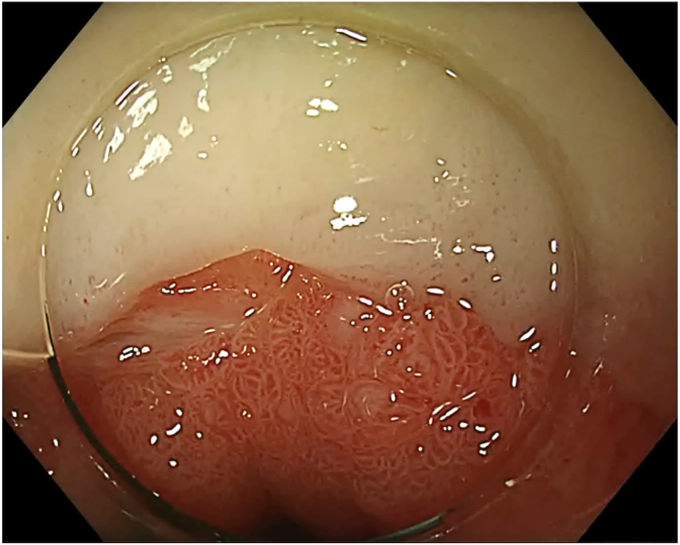
The fistula opening at the anastomosis, visualized by endoscopy.

**Figure 4:**
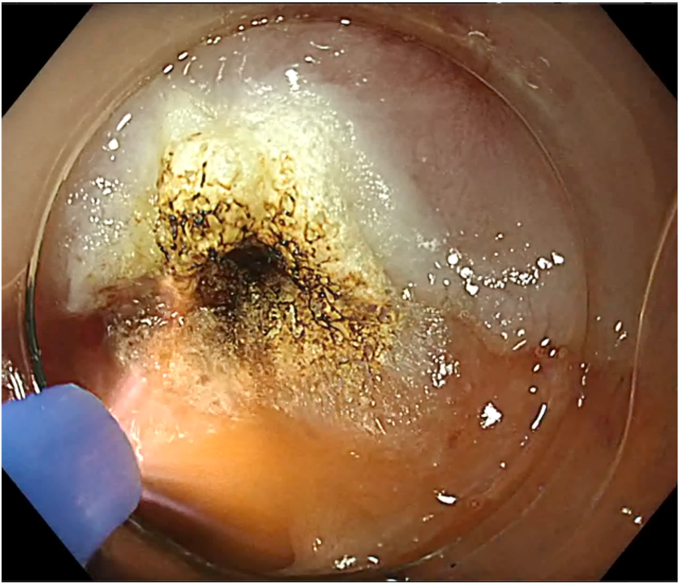
Argon plasma coagulation of the fistula edges.

**Figure 5:**
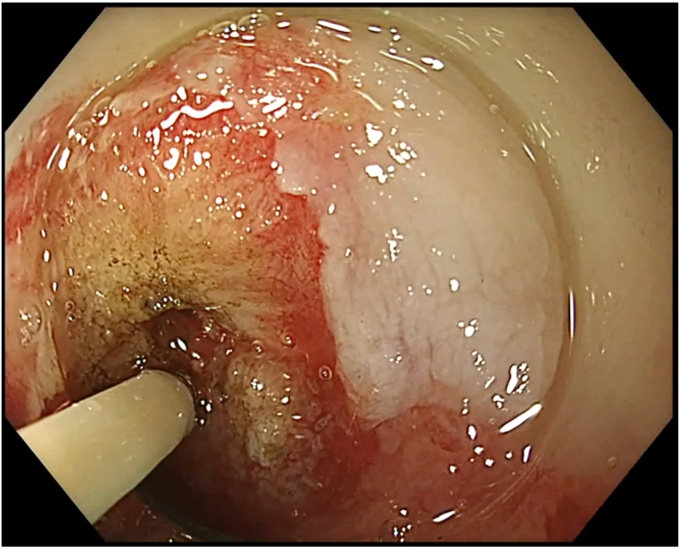
Injection of the stromal vascular fraction and platelet-rich plasma mixture around the fistula opening.

**Figure 6:**
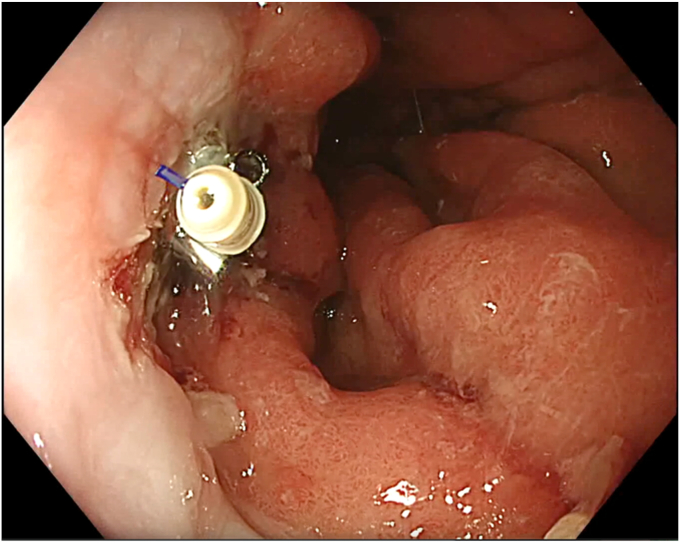
The fistula opening was closed with the X-tack system (Boston Scientific, Marlborough, Mass, USA).

During SVF isolation, endoscopy (in 1 case combined with bronchoscopy) was performed to visualize the BEF (Fig. 3). The fistula tract was brushed with a cytology brush to create a vital wound surface. Argon plasma coagulation (pulsed, 40 W) of the fistula edges was performed at the esophageal side (Fig. 4). Once the fistula tract was cleaned, the mixture of PRP with SVF was injected around the fistula orifices (Fig. 5). Because of the viscosity of the injectate, a 19-gauge FNA needle was used in conjunction with 1-mL Luer-lock syringes to facilitate injection. In the patient with a fistula to the trachea, the entire procedure was bronchoscopically controlled to ensure proper placement of the PRP and SVF mixture (Video 1, available online at www.videogie.org). Finally, the fistula edges were approximately closed using an endoscopic through-the-scope suturing system (Boston Scientific, Marlborough, Mass, USA) (Fig. 6). Postoperatively, patients were kept nil per mouth, with enteral nutrition via jejunostomy that had been placed at the time of esophagectomy, until esophagram 1 month postprocedure confirmed fistula closure.

## Conclusion

Both patients recovered significantly with this minimally invasive procedure. They are on an oral diet without signs of aspiration and a good quality of life. Limitations of this procedure include the possibility that large fistulous openings with extensive fibrosis may not be amenable to closure, as the esophageal wall may lack sufficient pliability. In addition, there is a potential risk of injection of the SVF-PRP mixture into the trachea or endovascular, which could result in, respectively, pneumonitis/bronchitis or may lead to a fat embolism.

## Patient consent

Although the case was anonymized, the patient was informed about the publication and provided consent.

## Disclosure

The following authors disclosed financial relationships: R. E. Pouw: Consultant for Boston Scientific, MicroTech Europe, Cook Medical, and Medtronic BV; research support from Moeller Medical; honorarium from Fujifilm and Olympus; speaker for Pentax BV. J. A. van Dongen: Instructor for Arthrex. R. van Hillegersberg: Proctor for Intuitive Surgical; advisory board for Medtronic, Olympus, and Ethicon. All other authors disclosed no financial relationships.

## Supplementary data

Video 1 xxx
